# Biomechanical Research of Three Parallel Cannulated Compression Screws in Oblique Triangle Configuration for Fixation of Femoral Neck Unstable Fractures

**DOI:** 10.1111/os.14004

**Published:** 2024-02-22

**Authors:** Ru‐Yi Zhang, Wu‐Peng Zhang, Guang‐Min Yang, Dao‐Feng Wang, Peng Su, Yi Zhang, Shao‐Bo Nie, Jia Li, Zhe Zhao, Jian‐Tao Li, Li‐Cheng Zhang, Pei‐Fu Tang

**Affiliations:** ^1^ Department of Orthopaedics Shijingshan Teaching Hospital of Capital Medical University, Beijing Shijingshan Hospital Beijing China; ^2^ Department of Orthopedics The Fourth Medical Center of Chinese PLA General Hospital Beijing China; ^3^ National Clinical Research Center for Orthopedics, Sports Medicine & Rehabilitation Beijing China; ^4^ School of Medicine, Nankai University Tianjin China; ^5^ Department of Orthopaedics Beijing Tsinghua Changgung Hospital, School of Clinical Medicine, Tsinghua University Beijing China; ^6^ Department of Sports Medicine Sports Medicine Service, Beijing Jishuitan Hospital, Capital Medical University Beijing China

**Keywords:** Biomechanics, Cannulated Compression Screw, Femoral Neck Fracture, Oblique Triangle, Spatial Configuration

## Abstract

**Objective:**

Surgical treatment with internal fixation, specifically percutaneous fixation with three cannulated compression screws (CCSs), is the preferred choice for young and middle‐aged patients. The mechanical advantage of the optimal spatial configuration with three screws provides maximum dispersion and cortical support. We suspect that the spatial proportion of the oblique triangle configuration (OTC) in the cross‐section of the femoral neck isthmus (FNI) may significantly improve shear and fatigue resistance of the fixed structure, thereby stabilizing the internal fixation system in femoral neck fracture (FNF). This study aims to explore the mechanical features of OTC and provide a mechanical basis for its clinical application.

**Methods:**

Twenty Sawbone femurs were prepared as Pauwels type III FNF models and divided equally into two fixation groups: OTC and inverted equilateral triangle configuration (IETC). Three 7.3 mm diameter cannulated compression screws (CCSs) were used for fixation. The specimens of FNF after screw internal fixation were subjected to static loading and cyclic loading tests, respectively, with five specimens for each test. Axial stiffness, 5 mm failure load, ultimate load, shear displacement, and frontal rotational angle of two fragments were evaluated. In the cyclic loading test, the load sizes were 700 N, 1400 N, and 2100 N, respectively, and the fracture end displacement was recorded. Results were presented as means ± SD. Data with normal distributions were compared by the Student's *t* test.

**Results:**

In the static loading test, the axial stiffness, ultimate load, shear displacement, and frontal rotational angle of two fragments were (738.64 vs. 620.74) N/mm, (2957.61 vs. 2643.06) N, (4.67 vs. 5.39) mm, and (4.01 vs. 5.52)° (*p* < 0.05), respectively. Comparison between the femoral head displacement after 10,000 cycles of 700N cyclic loading and total displacement after 20,000 cycles of 700–1400N cyclic loading showed the OTC group was less than the IETC group (*p* < 0.05). A comparison of femoral head displacement after 10,000 cycles of 1400N and 2100N cycles and total displacement after 30,000 cycles of 700–2100N cycles showed the OTC group was less than another group, but the difference was not significant (*p* > 0.05).

**Conclusion:**

When three CCSs are inserted in parallel to fix FNF, the OTC of three screws has obvious biomechanical advantages, especially in shear resistance and early postoperative weight‐bearing, which provides a mechanical basis for clinical selection of ideal spatial configuration for unstable FNF.

## Graphical abstract

In this study, 20 fourth‐generation Sawbone femurs were used to evaluate the fixation mechanics of two screw space configurations (OTC triangle configuration and IETC inverted equilateral triangle configuration) for femoral neck fractures. During the experiment, static load test and cyclic load test were carried out on FNF specimens after internal screw fixation. The evaluation indexes of the static load test were axial stiffness, 5 mm failure load, ultimate load, shear displacement and front rotation angle of the two fragments. The cyclic loading test loads were 700N, 1400N, and 2100N, respectively, and the evaluation index was fracture end displacement. The study verified the mechanical advantages of OTC through *in vitro* biomechanical experiments, and to provide mechanical basis for its clinical application.

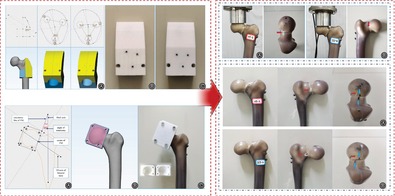

## Introduction

The selection of a treatment plan needs to consider various factors such as the patient's age, fracture type, overall physical condition, actual activity capability, and anticipated functional requirements. And the selection of surgical treatment for unstable FNF is always a clinical question. The internal fixation technique of femoral neck fracture has been continuously improved and developed in recent years, and hip‐preserving treatment mainly based on internal fixation is still the optimal choice for young and middle‐aged people with femoral neck fracture. Percutaneous fixation with three cannulated compression screws (CCSs) among current fixations is still the priority.^1^, ^2^, ^3^, ^4^ At present, the hot issue of three screws fixation of FNF is not only the configuration problem but also the cortical support. Therefore, many experts and scholars suggest that the placement of three screws should follow four conditions of parallel, inverted triangle, scattered, and cortical support at the same time.^5^, ^6^ Currently, the optimal spatial configuration (oblique triangle configuration, OTC) is the distribution of three CCSs for parallel fixation of FNF in an inverted triangle, which simultaneously satisfies the maximum degree of dispersion and obtains the maximum cortical support. Finite element analysis has confirmed that the mechanical stability of three screws in oblique triangle (OT) fixation of FNF is significantly superior to the traditional inverted equilateral triangle fixation.^7^ However, no relevant biomechanical studies have been reported so far.

According to the mathematical theory, it is known that the oblique triangle with fixed vertex (femoral calcar screw) occupies the largest area and has a constant ratio to the area of the ellipse. Therefore, it is theoretically analyzed that the three parallel screws in OTC have the largest occupying effect and can achieve the advantage of fixation stability when fixing femoral neck fractures. As a result, medical professionals are currently investigating the subsequent iteration of efficacious configuration for CCSs fixation. The purpose of this study was to verify the mechanical advantages of OTC through *in vitro* biomechanical experiments and to provide the mechanical basis for its clinical application. We hypothesized that the utilization of OTC would yield enhanced biomechanical stability in comparison to IETC, owing to its superior anchoring capabilities and angular stability. We assert that the present investigation has the potential to establish a universally accepted approach for the development of configuration design in the domain of biomechanical study of unstable femoral neck fractures.

## Materials and Methods

### Study Design

Twenty fourth‐generation Sawbones femurs (Model 3406, 17 PCF, Solid Foam Core, Large, LEFT; Sawboness, Vashon, WA) were selected. For simulated cortical bone (short fiber‐filled epoxy), the density is 1.64 g/cm^3^. The longitudinal tensile strength is 106 MPa, and the longitudinal tensile modulus is 16.0 Gpa. The transverse tensile strength is 93 Mpa, and the transverse tensile modulus is 10.0 Gpa. The compressive strength is 157 Mpa, and the compressive modulus is 16.7 Gpa. For simulated cancellous bone (rigid polyurethane foam), the density is 0.32 g/cm^3^. The strength is 106 Mpa, and the modulus is 16.0 Gpa. They were equally divided into two groups, OTC group and inverted equilateral triangle configuration (IETC) group. The two groups were subjected to static loading test and cyclic loading test, respectively, with five specimens in each test (Figure S1).

### Computer‐Aided Design (CAD)

**Figure 1:**
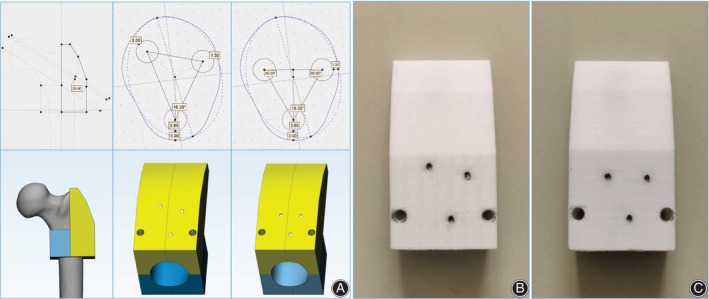
The CCS guide pin (diameter: 2.5 mm) placed guide plate was designed by Mimics software and 3D‐printed. (A). guide plate was designed by Mimics software, wherein the Sawbones used in the experiment had the femoral neck torsional angle of 16.50. (B). OTC guide plate, C. IETC guide plate.

**Figure 2:**
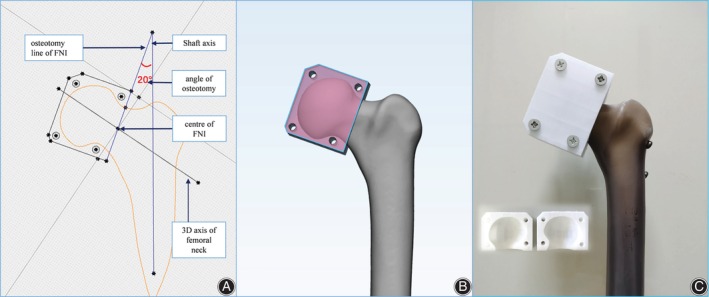
(A, B) The osteotomy guide plate through the femoral neck isthmus was designed by Mimics software. (C) The actual osteotomy guide plate was 3D printed.

**Figure 3:**
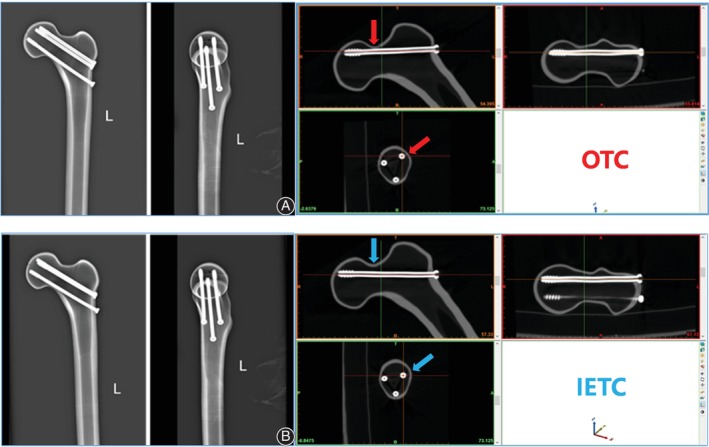
The anteroposterior and lateral X‐ray and CT images of a fixed femoral neck fracture model with CCSs in two configurations. (A) OTC. (B) IETC. The position of the femoral calcar screw in the two configurations was consistent. The OTC position of the posterior screw was slightly higher than that of the IET (indicated by the red and blue arrows respectively), and the OTC position of the anterior screw was significantly higher than that of the IETC. In OTC, three screws were attached to the femoral neck cortex, while in IETC, the anterior and superior screws were not attached to the femoral neck cortex, and did not achieve cortical support.

**Figure 4:**
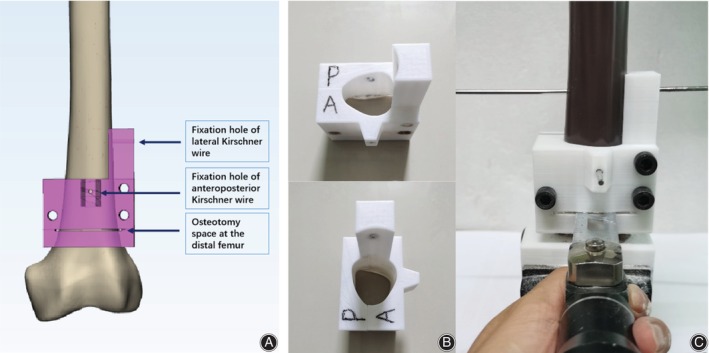
(A) The distal femur osteotomy guide plate was designed by Mimics software, (B) the actual distal femur osteotomy guide plate was 3D printed.

One sample was randomly selected for a 64‐slice spiral CT scan and reconstruction. The scan slice thickness was set to 5 mm. Then, the medical Digital Imaging and Communications in Medicine (DICOM) files of the selected CT scan were retrieved and loaded into Mimics software (Version 20.0, Materialize, Belgium), and the femur model was reconstructed. First, the guide pins (diameter 2.5 mm) of two spatial configurations (OTC and IETC) were designed to be inserted into the guide plate by using the method in our previous optimal configuration demonstration design to ensure the accuracy of CCS implantation and the uniformity of specimen preparation^8^ (Figure 1). Three guide wires were placed into the femoral head neck. A 7.3 mm diameter partially threaded cancellous bone screw of appropriate length was selected and placed in the femoral neck, followed by the withdrawal of three screws. Second, we firstly found the long axis plane of the femoral neck isthmus (FNI) according to the previous test method,^8^ and projected the long axis of the femoral model, femoral neck axis, and the long axis of isthmus cross section on the long axis plane of the femoral neck to obtain femoral head and neck outlines and the osteotomy line of the FNI (Pauwels Angle = 70°). 3D reconstruction in STL format was imported into 3‐Matic software (Version 12.0, Materialize, Belgium) for designing the osteotomy model. Then, the osteotomy guide plate and four fixing bolt holes were designed. Furthermore, the physical object was printed by 3D printing technology, and the anteroposterior part of guide plate was fixed on the femoral head and neck with four screws for osteotomy. To reduce the interference factors of the experiment, a widely reported single‐plane osteotomy was adopted (Figure 2).^9^, ^10^ Third, the three screws removed previously were screwed into the original position under fluoroscopy (Figure 3). The proximal of the femur was used for the mechanical test. The fourth step was to design the guide plate for distal femur osteotomy. The position 10 cm above the femur condyle was selected as the osteotomy plane, and the distal 10 cm was placed in an anatomical structure morphology‐matched fixation device equipped with a designed 1.6 mm oscillating saw seam and Kircher wires (Figure 4). The osteotomy and fixation of all femoral models were performed by the same deputy chief physician. The cross‐section of the femoral neck isthmus was obtained by osteotomy plane through guide plate. The area of the OT and the inverted equilateral triangle and the cross‐sectional area of the FNI were read directly in the software. The ratio of the area of the triangle to the cross‐section is the area proportion.

**Figure 5:**
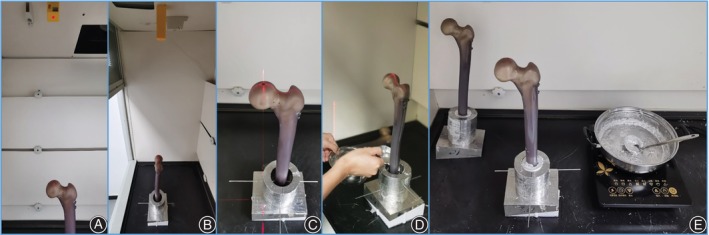
Embedding and fixation of the femoral model. (A–C) The position of the femoral model was standardized and unified before embedding. (D, E) The femur model was embedded with Wood alloy.

Wood alloy with satisfactory mechanical strength^11^ is used as the embedding material at distal femur. The “cross” red light positioning method was used to ensure the position consistency of the femoral model embedding (Figure 5). The fixtures (fixtures and bases for fixing the proximal and distal ends of the femoral model) were designed based on the morphology of Sawbones.^12^ The distal fixture has an inclination angle (10° adduction, 9° retrotilt), which simulates the force of the femoral head from the acetabulum when standing on one leg.

### Static and Cyclic Loading Tests

**Figure 6:**
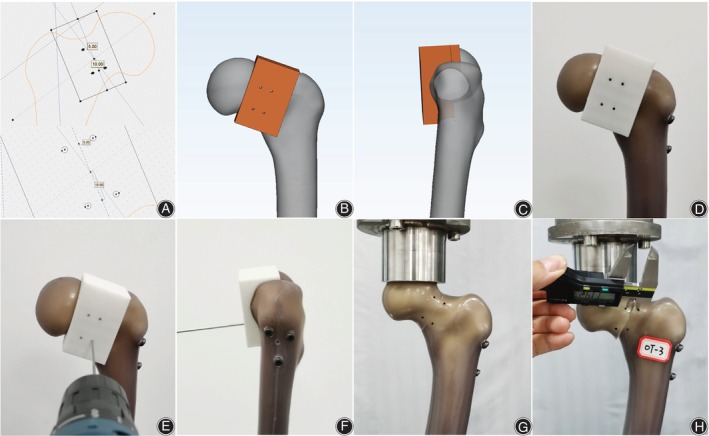
The location and measurement of marker points at the femoral neck fracture end (A–D). The design and 3D printing of two pairs of corresponding point guide plate on the upper and lower sides of fracture line in front of femoral neck (E–G). The marking of two corresponding points (H). the distance measurement between corresponding points under axial loading.

The static and cyclic loading tests were conducted. The former was used to evaluate the shear resistance of screw configurations. The 3D printed marker point guide plate was used to mark two pairs of corresponding points on the 5 mm sides of the upper and lower sides of the fracture line in front of the femoral neck, and black with a marker pen. The distance between the two points on the same side was 20 mm (Figure 6). The mechanical loading starts from 0N and vertically downward at a rate of 1 mm/min until the ultimate load appears.^13^ The experiment was terminated immediately when there was a serious fracture or bone destruction beyond the fracture line, internal fixation cut out, or internal fixation deformity.^14^ The experiment ending was marked by the upward trend of the force‐displacement curve becoming slow or a downward trend when it reached the peak. The evaluation indexes were the axial stiffness, 5 mm failure load, ultimate load, shear displacement, and frontal rotational angle of fracture ends. A vernier caliper was used to measure the initial displacement and the ultimate displacement directly above the fracture end, and the shear displacement of the fracture end was obtained by the difference between the two displacement values.

**Figure 7:**
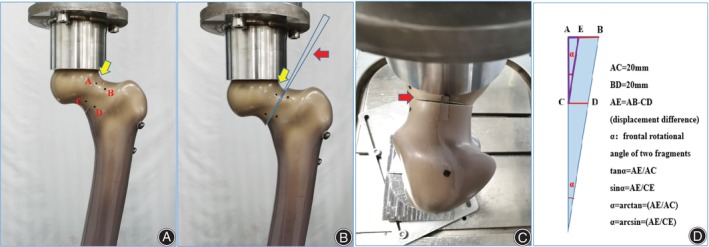
The measurement of shear displacement and frontal rotational angle of two fragments during static loading test. (A, B) Yellow arrows show the shear displacement of the femoral head and neck, measured with vernier calipers. (B, C) Red arrows show the fracture opening, (D) the calculation method of the frontal rotational angle α.

The frontal rotational angle of the two fragments was obtained indirectly by mathematical calculation. Two pairs of corresponding points were marked at 5 mm on the upper and lower sides of the fracture line in front of the femoral neck. When the ultimate load was reached, the distance between two pairs of corresponding points was measured respectively, and the change of distance between two points was calculated. We first calculated the Sin and Tan values of the frontal rotational angle respectively, and then compared them with the frontal rotational angles calculated by Arcsine and Arctangent methods (Figures 6 and 7).

**Figure 8:**
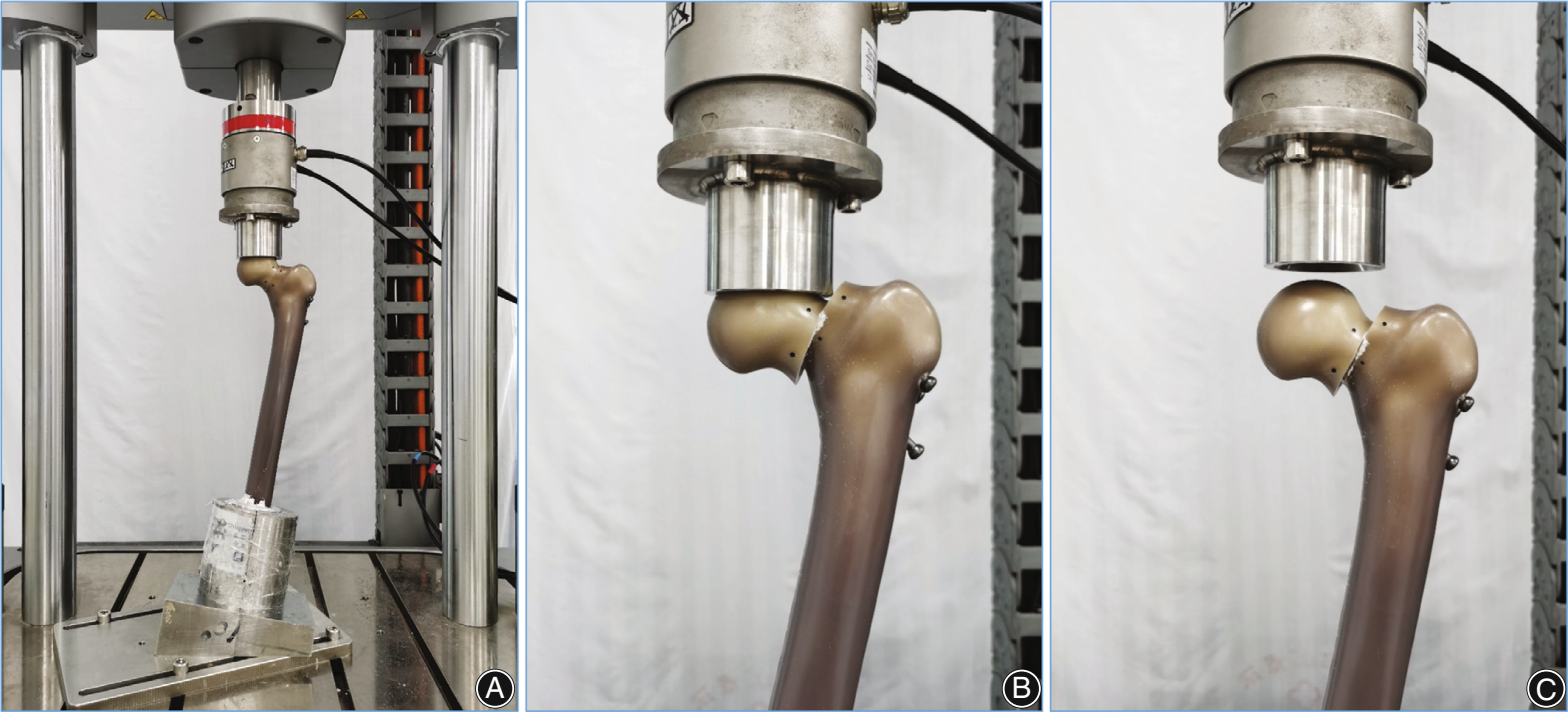
Cyclic loading test. (A) Prepare for loading after embedding. (B) Maintain loading under failure load. (C) Load removal after failure load.

A cyclic loading test was used to assess the fatigue resistance of two screw configurations. Referring to the load simulated weight‐bearing load of patients during postoperative rehabilitation in the previous study, the 70–700N, 140–1400N, and 210–2100N were vertically loaded down with sinusoidal waveform (load ratio was 0.1), and each was loaded 10,000 times at 3 Hz frequency (Figure 8), which simulate weight‐bearing walking 4–6 weeks early after surgery.^15^ The evaluation indexes were the femoral head displacement at 10000 cycles under different loadings, 700–1400N total displacement, and 700–2100N total displacement.

During the experiment, static load test and cyclic load test were carried out on FNF specimens after internal screw fixation. The evaluation indexes of the static load test were axial stiffness, 5 mm failure load, ultimate load, shear displacement, and front rotation angle of the two fragments. The cyclic loading test loads were 700N, 1400N, and 2100N, respectively, and the evaluation index was fracture end displacement.

### Statistical Analysis

All statistical analyses were performed using SPSS 21.0 (IBM, Armonk, NY, USA). Values are presented as the mean ± standard deviation for data that were normally distributed. For two‐group comparison, *p* values were derived from the one‐way student *t*‐test to compare the difference between the static loading test results and cyclic loading test results of two configurations. The significance level for all statistical tests was set at *p* < 0.05.

## Results

The area ratio of the OT and the inverted equilateral triangle (IET) in the isthmus cross‐section of the femoral neck in two configurations showed that the area proportion of OT was greater than that of IET (Table 1).

**Table 1:** The proportion of the OT and IET in the cross‐section of the femoral neck isthmus.

	OT	IET	Cross‐section
Area (mm^2^)	393.79	359.53	958.27
Ratio	41.09%	37.52%	100%

### Static Loading Test

**Figure 9:**
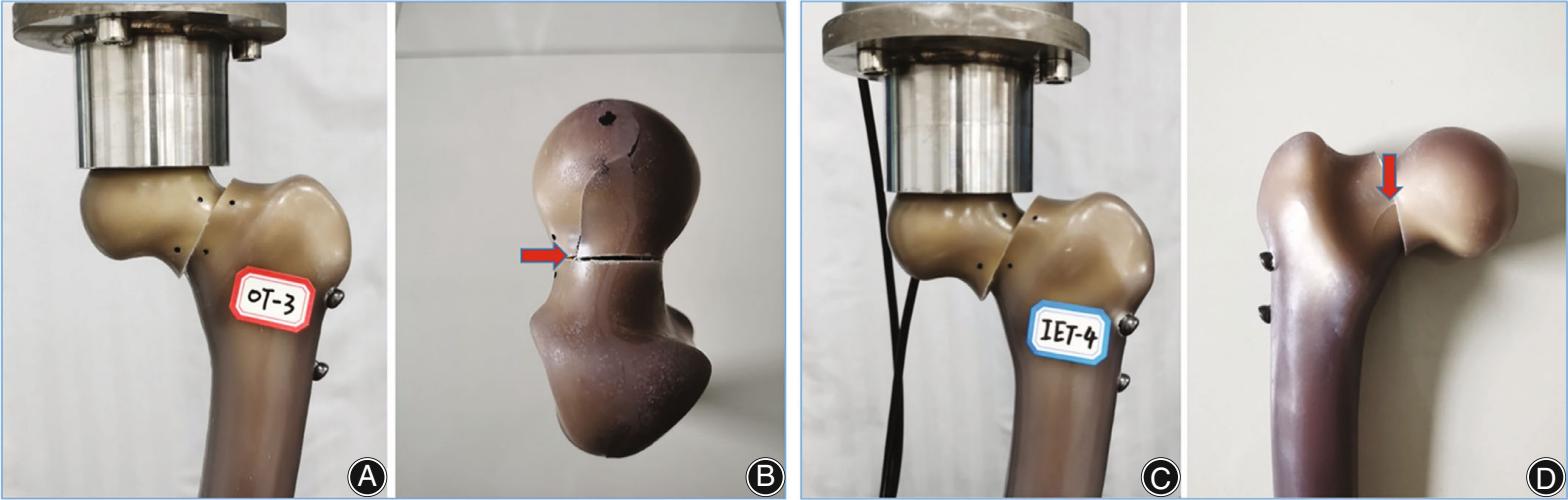
Shear displacement and slight frontal rotational angle of two fragments were observed in both groups of models during loading. (A) In the OTC group, splitting occurred in the anterior and superior cortex bone of the proximal femoral neck fracture fragment. (B) In the IETC group, splitting occurred in the posterior cortex bone of the distal femoral neck fracture fragment.

In the OTC and IETC groups, shear displacement gradually occurred along the fracture section at the proximal fracture fragment as the load gradually increased, followed by slight separation above the fracture section and slight angulation at the fracture end. However, there was no axial rotation of the proximal fracture fragment in both groups. With the advent of limit loads, the femoral neck anterior superior cortex of the proximal fracture fragment in all OTC samples split and extended to the femoral head, while the posterior superior cortex and distal inferior cortex of the proximal fracture fragment were intact. In all specimens of the IETC group, the posterior cortex of the distal femoral neck fracture fragment was split, while the superior cortex of the proximal fracture fragment and the inferior cortex of the distal fracture fragment were intact (Figure 9).

**Figure 10:**
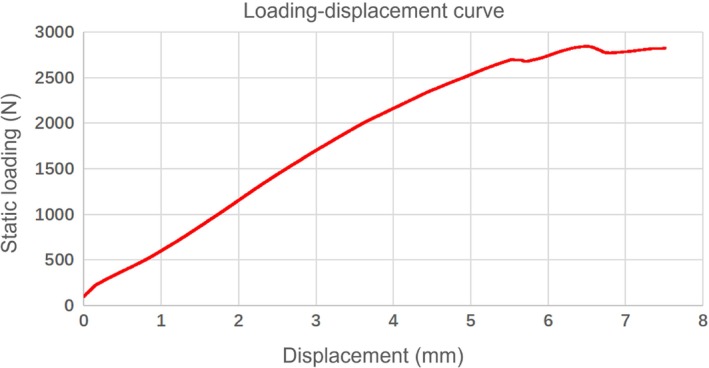
The stress–strain curve for axial static loading of IETC group.

Intra‐group comparison showed that there was little difference in the axial stiffness, 5 mm failure load, and ultimate load. In terms of axial stiffness and ultimate load, the OTC group was significantly larger than the IETC group (*p* < 0.05). And, in terms of 5 mm failure load, the OTC group was also larger than the IETC group, but the difference was not statistically significant (*p* > 0.05) (Table 2). Figure 10 has illustrated the stress–strain curve for axial static loading of IETC group, and the magnitude of displacement was used to indicate the magnitude of strain in this study. The intra‐group comparison showed that there was little difference in the shear displacement of fracture end, the difference between two points (the distance between AB and CD), and the frontal rotational angle of two fragments between the two groups. Compared between groups, these three indicators in the OTC group were significantly smaller than those in the IETC group (*p* < 0.05) (Table 3).

**Table 2:** Comparison of static loading test results of two configurations (axial stiffness and load).

Configuration	Axial stiffness (N/mm)	5 mm failure load (N)	Ultimate load (N)
OTC	738.64 ± 32.88	2891.33 ± 183.96	2957.61 ± 149.09
IETC	620.74 ± 72.74	2589.70 ± 237.88	2643.06 ± 238.05
*p* value	0.011	0.055	0.037
*t* value	3.303	2.244	2.504

**Table 3:** Comparison of static loading test results of two configurations (displacement and frontal rotational angle of two fragments).

	SD (mm)	DD (mm)	tan α /sin α	α = arctan (°)	α = arcsin (°)
OTC	4.67 ± 0.41	1.41 ± 0.19	0.07 ± 0.01	4.01 ± 0.54	4.02 ± 0.55
IETC	5.39 ± 0.36	1.93 ± 0.19	0.10 ± 0.01	5.52 ± 0.55	5.55 ± 0.56
*p* value	0.018	0.002	0.002	0.002	0.002
*t* value	2.962	4.373	4.373	4.373	4.373

*Note*: α: frontal rotational angle of two fragments; DD, displacement difference between two points; SD, shear displacement.

### Cyclic Loading Test

**Figure 11:**
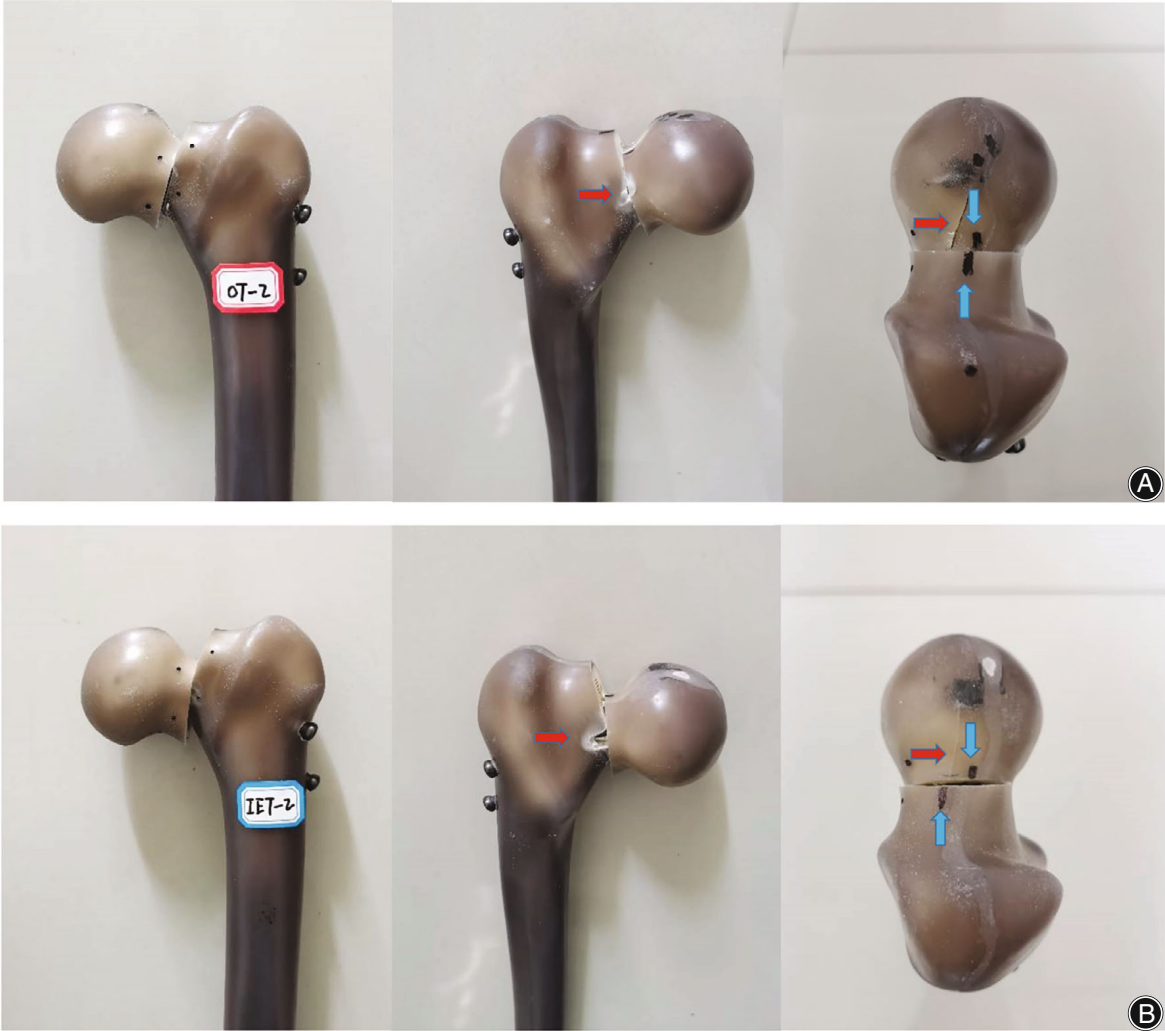
Appearance of the two groups of specimens after the 2100N cycle loading. (A) In the OTC group, the posterior cortex bone of the distal femoral neck fracture fragment was damaged slightly (red arrow), while significant splitting of the anterior and superior cortex bone of the proximal femoral neck fracture fragment (red arrow) and slight rotation of the femoral head backward (blue arrow). (B) In the IETC group, the posterior cortex bone of the distal femoral neck fracture fragment was severely damaged (red arrow), while the anterior and superior cortex bone of the proximal femoral neck fracture fragment was slightly split (red arrow) and the femoral head rotated backward apparently (blue arrow).

The two groups (OTC group and IETC group) of fracture models after internal fixation completed 10,000 cycles in each of the three loads. In the process of 700N and 1400N cyclic loading and the early stage of 2100N cyclic loading, the shear displacement and the frontal rotational angle of two fragments in the OTC group and the IETC group were roughly the same. During the 2100N cycle, the three screws in the OTC group were probed with hollow guide pins, and the bending stress was found to be almost simultaneous. Screw loosening and withdrawal were observed at the late stage of the cycle (the inferior screw had the largest withdrawal distance). In the IETC group, the bending stress of three screws was found in sequence during the 2100N cycle, the bending sequence was the inferior, the posterior, and the anterior. And the loosening and withdrawal of screws were observed (also, the inferior screw had the largest withdrawal distance). In the OTC group, the shear displacement of the proximal fracture fragment along the fracture section and the frontal rotational angle between the two fragments increased significantly at the end of the 2100N cycle, and mild femoral head rotation was observed. In the IETC group, posterior cortical splitting of the distal fracture fragment was observed, and significant femoral head rotation was observed backward (Figure 11).

Intra‐group comparison showed that there was little difference in cyclic loading test results. The shear displacement of the OTC group was smaller than that of the IETC group after 10,000 cycles of 700N, 1400N, and 2100N loading. There was a statistically significant difference between the two groups under 700N cyclic loading (*p* < 0.05), but no significant difference between the two groups under 1400N and 2100N cyclic loading (*p* > 0.05). The comparison of total displacement after 700–1400N and 700–2100N cyclic loading showed that the OTC group was smaller than the IETC group, and the difference in total displacement during 700–1400N cyclic loading was statistically significant (*p* < 0.05), while the difference of total displacement after 700–2100N cyclic loading was not statistically significant (*p* > 0.05) (Table 4).

**Table 4:** Comparison of cyclic loading test results of two configurations (displacement: mm).

Configuration	700 N	1400 N	2100 N	700–1400 N	700–2100 N
OTC	0.34 ± 0.03	0.77 ± 0.13	1.21 ± 0.15	1.70 ± 0.09	3.91 ± 0.83
IETC	0.47 ± 0.05	0.91 ± 0.08	1.59 ± 0.39	1.96 ± 0.20	5.68 ± 1.83
*p* value	0.001	0.080	0.077	0.028	0.084
*t* value	4.936	2.001	2.026	2.688	1.969

## Discussion

The static loading test revealed notable distinctions in the axial stiffness, ultimate load, shear displacement, and frontal rotation angle between the two fragments. These distinctions were observed as (738.64 vs. 620.74) N/mm, (2957.61 vs. 2643.06) N, (4.67 vs. 5.39) mm, and (4.01 vs. 5.52)°, respectively. Moreover, the comparison between the displacement of the femoral head after 10,000 cycles of 700N cyclic loading and the total displacement after 20,000 cycles of 700–1400N cyclic loading demonstrated that the OTC group exhibited lower displacement compared to the IETC group (*p* < 0.05).

### Predetermined Position

The model's fixed position would directly affect the relevant mechanical experimental results in both axial loading and cyclic loading tests. Some authors chose 13° adduction and 8° flexion as the fixed positions of specimens,^16^, ^17^ believing the angle between the resultant force of the hip upon the femoral head and the central vertical line on the coronal plane of the body is about 12°–16°^18^ in daily activity. Our team selected fixed positions of 10° adduction and 9° retrotilt, which better simulated the real situation of stress on the femoral head of normal people standing on one leg.^19^, ^20^ At the same time, we believe that no matter what position specimens are selected before loading, as long as strict and consistent experimental conditions are guaranteed, the influence of non‐experimental factors on the test can be reduced, the baseline of experimental subjects is consistent, and the reliability of results can be improved.

### Parameters Setting

For the setting of mechanical parameters, the static loading test adopts our team's standard.^12^ However, the load size during cyclic loading has not been strictly defined at present, most scholars prefer to carry out loading under the multiple of standard weight. For example, Selvan and Rupprecht used 700–750 N loads to simulate partial weight‐bearing after surgery,^21^, ^22^ Springer and Yang used 1300–1400 N loads to simulate two times body weight,^14^, ^23^ Hawks used 2100N loads to simulate three times body weight.^24^ We believe that the comprehensive consideration of the three loads is closer to the guidance of clinical postoperative rehabilitation.

### Primary Outcomes

In this study, the shear displacement of the fracture end, the distances between the two pairs of corresponding points, and the frontal rotational angle under the ultimate load were increased. The shear resistance and stability of the internal fixation system were compared directly by measuring and comparing the shear displacement of the fracture end and the actual frontal rotational angle of the fracture end under the ultimate load. The frontal rotational angle of two fragments is not very large in practical operation, especially in the case of a small loading force, so it is difficult to measure and evaluate mechanically. In this study, the actual frontal rotational angle was calculated by measuring the distances between two pairs of corresponding points. The specific method is to calculate the sine and tangent values of the frontal rotational angle, respectively, and then calculate the frontal rotational angle by arcsine and arctangent. There is no obvious difference between the three values.

In static loading test, the axial stiffness, 5 mm failure load, and ultimate load of the OTC group were higher than the IETC group, on the contrary, the shear displacement and the frontal rotational angle of the OTC group were lower than the IETC group. Except for the 5 mm failure load, the differences of the other four observation indexes between groups were statistically significant, which fully confirmed that the shear resistance of the model of Pauwels III FNF fixed with three parallel OTC screws was superior to that of IETC. The results show that there are differences in locations of bone structure splitting when the loading reaches the ultimate load. In the OTC group, the split was located in the anterior superior femoral neck cortex bone of the proximal fracture fragment and extended over the femoral head, while in the IETC group, the split occurred behind the distal fracture fragment. These results suggest that the two fixation methods show different fixation efficiency.

Three screws being used to fix Pauwels type III FNF with parallel OTC fixation has certain advantages over IETC fixation in partial weight‐bearing and early functional rehabilitation. In addition, we found that the three screws in OTC group showed a tendency to bend almost simultaneously, and the backward rotation angle of the femoral head was significantly smaller than that of the IETC group during the middle and late period of the 2100N load cycle, suggesting that the three screws in the OTC group obtained cortical support at the same time and had a potential anti‐rotation advantage.

Three parallel OTC screws had obvious mechanical advantages in shear and fatigue resistance for the Pauwels type III FNF model. Tang et al. have put forward the theory of triangular mechanics of hip fracture.^25^, ^26^ The basis of this theory is to equate the structural factors maintaining hip stability to a triangular mechanical stability mechanism constructed by the medial, lateral, and upper wall through mechanical derivation.^12^ Theoretical mechanical analysis of the role of each side in maintaining the stability of the proximal femur^27^: (1) the medial wall forming the oblique support of the proximal femoral cantilever structure greatly reduced the bending stress and deflection of the structure, (2) the lateral wall could effectively reduce the sliding and deflection of the femoral neck under physiological loads, (3) the upper wall acted as a connection between the medial and lateral edges of the proximal femur, making the medial and lateral structures of the proximal femur resist the bending moment caused by physiological loads. We believe that OTC can reconstruct the upper and medial wall of the hip better than IETC. Because the upper two screws of the OTC are more suitable to the upper femoral neck section, the upper lateral structure get well rebuilt. And the lower screw provides medial cortical support and reconstructs the medial wall. It is an important reason for OTC to reconstruct the triangular mechanics of hip and get the biomechanical advantage.

### Strengths and Limitations

The development of composite bone models has made it possible to achieve a high degree of fidelity to human anatomy with similar biomechanical properties, which can be used as a substitute for cadaveric bones. This will ensure the reproducibility of the surgical technique. Based on the biomechanical advantages demonstrated by the OTC group, screws with oblique triangular configuration have biomechanical advantages of shear and fatigue resistance in the fixation of femoral neck fracture, which is a suitable choice for the treatment of unstable femoral neck fracture. Meanwhile, to achieve the optimal insertion position for the screws in the OTC group during clinical surgery, a robotic‐assisted screw placement approach can be employed. This method not only maximizes cortical support of the femoral neck but also helps to avoid the “in‐out‐in” situation of the upper‐posterior one screw during the screw placement process in fluoroscopic navigation. This, in turn, reduces the risk of damage to the lateral circumflex femoral arteries and the potential for femoral head necrosis.

Although the oblique‐triangle configuration method is still in its initial stages, there are currently important validation studies being conducted on patients who are receiving treatment for femoral neck fractures. One major limitation of the study is that the sample size may not be sufficient to divide the composite bones into two groups for statistical analysis of relevant parameters. Additionally, composite bone models with highly consistent morphology exhibit biomechanical properties that are highly congruent with human anatomy, but they are still not a perfect substitute for cadaveric bone. It is imperative to conduct testing prior to clinical translation, when conditions allow, using cadaver bone models to confirm the accuracy and feasibility of this method. For unstable femoral neck fractures, there have been studies comparing the biomechanical properties of the femoral neck system and cannulated screws, but there is a lack of studies on OTC.^28^, ^29^ This outcome necessitates additional validation through further clinical investigations.

## Conclusion

In this study, we compared the oblique‐triangle configuration (OTC) and inverted equilateral triangle configuration (IETC) in terms of their mechanical performance. The OTC demonstrated enhanced mechanical characteristics when compared to the IETC in experimental models utilizing Sawbone femurs. The findings of this investigation imply that employing a parallel fixation technique with three screws may lead to improved stability of the internal fixation when utilizing larger cross‐sectional screw configurations. It is envisaged that the oblique‐triangle configuration could potentially reduce the risk of fracture fixation failure and reoperation in young and middle‐aged patients with FNF.

## CONFLICT OF INTEREST STATEMENT

There are no conflicts of interest of all authors.

## ETHICS STATEMENT

This is a biomechanical experiment based on Sawbones without ethical approval.

## AUTHOR CONTRIBUTIONS

All authors had full access to the data in the study and take responsibility for the integrity of the data and the accuracy of the data analysis. Conceptualization: Pei‐Fu Tang and Li‐Cheng Zhang. Methodology: Zhe Zhao, Ru‐Yi Zhang. Investigation: Jian‐Tao Li, Dao‐Feng Wang, Peng Su. Formal Analysis: Shao‐Bo Nie, Jia Li, Yi Zhang. Writing—Original Draft: Ru‐Yi Zhang, Wu‐Peng Zhang and Guang‐Min Yang. Writing—Review & Editing: Li‐Cheng Zhang. Supervision: Pei‐Fu Tang. All authors read and approved the final manuscript.

## AUTHORSHIP DECLARATION

All authors listed meet the authorship criteria according to the latest guidelines of the International Committee of Medical Journal Editors, and all authors are in agreement with the manuscript.

## Supporting information


**Figure S1.** The overview of groupings of biomechanical test.
