# Supplementary figures and images for: Biomechanical Research of Three Parallel Cannulated Compression Screws in Oblique Triangle Configuration for Fixation of Femoral Neck Unstable Fractures
Source: Orthop Surg. 2024 Feb 22;16(4):953–64. doi: 10.1111/os.14004 (PMC10984827; doi:10.1111/os.14004)

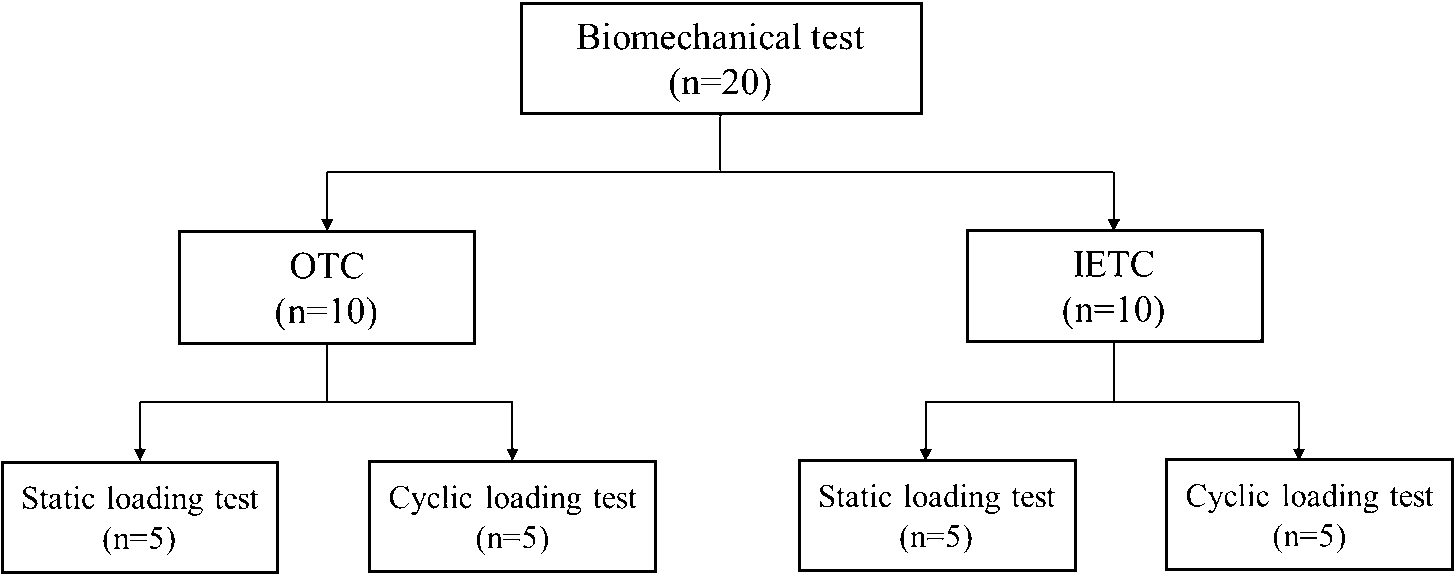

Supplement: Supplementary file 1 — Figure S1. The overview of groupings of biomechanical test. [file OS-16-953-s001.tif]
